# *Chrysanthemum morifolium* Ramat extract and probiotics combination ameliorates metabolic disorders through regulating gut microbiota and PPARα subcellular localization

**DOI:** 10.1186/s13020-024-00950-w

**Published:** 2024-06-03

**Authors:** Xinxin Gao, Zhigang Zhu, Yiyang Bao, Yifan Li, Weize Zhu, Xiaofang He, Xinyu Ge, Wenjin Huang, Hao Wang, Wenjing Wei, Jun Du, Liang Chen, Houkai Li, Lili Sheng

**Affiliations:** 1 School of Pharmacy, Shanghai University of Traditional Chinese Medicine, Shanghai, 201203 China; 2 Nutrilite Health Institute, Amway (Shanghai) Innovation & Science Co, Ltd, Shanghai, 201203 China

**Keywords:** *Chrysanthemum morifolium* Ramat, Probiotics, NAFLD, Gut microbiota, PPARα, T2DM

## Abstract

**Background:**

*Chrysanthemum morifolium* Ramat, a traditional Chinese medicine, has the effects on liver clearing, vision improving, and anti-inflammation. *C. morifolium* and probiotics have been individually studied for their beneficial effects on metabolic diseases. However, the underlying molecular mechanisms were not completely elucidated. This study aims to elucidate the potential molecular mechanisms of *C*. *morifolium* and probiotics combination (CP) on alleviating nonalcoholic fatty liver disease (NAFLD) and the dysregulation of glucose metabolism in high-fat diet (HFD)-fed mice.

**Methods:**

The therapeutic effect of CP on metabolism was evaluated by liver histology and serum biochemical analysis, as well as glucose tolerance test. The impact of CP on gut microbiota was analyzed by 16S rRNA sequencing and fecal microbiota transplantation. Hepatic transcriptomic analysis was performed with the key genes and proteins validated by RT-qPCR and western blotting. In addition, whole body *Pparα* knockout (*Pparα*^−/−^) mice were used to confirm the CP-mediated pathway.

**Results:**

CP supplementation ameliorated metabolic disorders by reducing body weight and hepatic steatosis, and improving glucose intolerance and insulin resistance in HFD fed mice. CP intervention mitigated the HFD-induced gut microbiota dysbiosis, which contributed at least in part, to the beneficial effect of improving glucose metabolism. In addition, hepatic transcriptomic analysis showed that CP modulated the expression of genes associated with lipid metabolism. CP downregulated the mRNA level of lipid droplet-binding proteins, such as *Cidea* and *Cidec* in the liver, leading to more substrates for fatty acid oxidation (FAO). Meanwhile, the expression of CPT1α, the rate-limiting enzyme of FAO, was significantly increased upon CP treatment. Mechanistically, though CP didn’t affect the total PPARα level, it promoted the nuclear localization of PPARα, which contributed to the reduced expression of *Cidea* and *Cidec*, and increased expression of CPT1α, leading to activated FAO. Moreover, whole body PPARα deficiency abolished the anti-NAFLD effect of CP, suggesting the importance of PPARα in CP-mediated beneficial effect.

**Conclusion:**

This study revealed the hypoglycemic and hepatoprotective effect of CP by regulating gut microbiota composition and PPARα subcellular localization, highlighting its potential for therapeutic candidate for metabolic disorders.

**Supplementary Information:**

The online version contains supplementary material available at 10.1186/s13020-024-00950-w.

## Introduction

Metabolic disorders, such as non-alcoholic fatty liver disease (NAFLD), type 2 diabetes mellitus (T2DM), obesity, and dyslipidemia, is increasingly endangering human health [1]. The incidence of metabolic disorders has been rapidly increased in recent years [2]. Inadequate metabolic management can lead to serious long-term complications. Due to the complexity and heterogeneity of the pathogenesis and progression of NAFLD, for a long time, drugs for treating NAFLD have been extremely scarce [3, 4]. Recently, the U.S. Food and Drug Administration approved Resmetirom, the first medication to treat nonalcoholic steatohepatitis (NASH) and liver fibrosis, suggesting targeted activation of essential receptors might be a possibility to treat NAFLD.

Gut microbiota and its metabolites play essential role in regulating host metabolism. Gut microbiota dysbiosis is associated with metabolic disorders such as NAFLD and T2DM [5]. Interventions like probiotics, fecal microbiota transplantation (FMT), and diet have shown potential in reshaping the gut microbiota to treat metabolic diseases such as NAFLD and T2DM [6]. FMT from health donors to T2DM patients significantly improved the clinical indicators like HOMA-IR and BMI of T2DM patients [7]. *Bifidobacterium lactis* and *Lactobacillus rhamnosus* have been shown to play an important role in host metabolic health [8, 9]. For example, *B. lactis* BPL-1, *L. rhamnosus* CNCMl-4036, *L. rhamnosus* BPL1-15 (CECT 8361), and *L. rhamnosus* GG have been reported to improving obesity, reducing inflammation, exerting antioxidant effects, and regulating gut microbiota, separately [10–13] Therefore, in current study, a combination of these four probiotics (a commercial product), was selected in the hope of combining the characteristics of each strain to explore their mechanism for improving metabolic diseases. Traditional Chinese Medicine (TCM) has been demonstrated to treat many metabolic diseases effectively. As one of the TCMs, *Chrysanthemum morifolium* Ramat, has the effects on liver clearing, vision improving, and anti-inflammation. The ethanol extract from *C. morifolium* leaves has been shown to prevent obesity and hepatic lipid degeneration in diet-induced obese mice [14]. Luteolin, a major component of *C. morifolium* extract, has been extensively studied for its anti-inflammatory, antibacterial effects, and its ability to alleviate LPS-induced liver damage by inhibiting oxidative stress and inflammation [15]. In addition, ethanol extract from *C. morifolium* flowers has been shown to inhibit the growth of pathogenic bacteria in *vitro* [16], while the aqueous extract from the flower heads of *C. morifolium* displayed its anti-inflammation effect in macrophages [17]. However, there is a lack of studies on their impacts on gut microbiota, and the function of aqueous extract from *C. morifolium* flowers on glucose metabolism is unknown. Although probiotics and *C. morifolium* showed beneficial effect on host health, the underlying mechanism is still largely unknown. Thus, in this study, we investigated the effect of the aqueous extract of *C. morifolium* flowers and probiotics (CP) on improving lipid and glucose metabolism in diet-induced NAFLD mouse model and explored the underlying mechanism.

Hepatic peroxisome proliferators activate receptor α (PPARα) is an essential transcriptional regulator in regulating fatty acid transport and fatty acid oxidation (FAO) in nucleus [18]. The peroxisome proliferator-activated receptor γ coactivator 1-α (PGC1α), a transcriptional coactivator induced by fasting [19, 20], can bind with PPARα to form heterodimer to regulate the expression of genes involved in lipid metabolism [21]. It has been reported that hepatocyte-specific PPARα knockout mice exhibit accelerated hepatic lipid accumulation [22]. Carnitine palmitoyltransferase 1α (CPT1α), the rate-limiting enzyme of FAO, has emerged as an important therapeutic target for NAFLD [23]. In addition, the apoptosis-induced DNA fragmentation factor 45-like effector (CIDE) proteins, including CIDEa and CIDEc, whose expressions are negatively regulated by PPARα, play a role in maintaining lipid homeostasis in adipose tissue and liver [24]. Previous studies have shown that liver specific knockout of *Cidea* can reduce liver lipid drop size and liver TG in *ob/ob* mice [25]. Thus, discovery of novel treatment approach targeting PPARα/PGC1α and CPT1α-mediated FAO is essential for maintaining the balance of fatty acid metabolism [26].

In this study, we found CP intervention alleviated lipid and glucose metabolism disorders in mice caused by high fat diet (HFD) feeding. CP improved glucose homeostasis through regulating gut microbiota, while the anti-NAFLD effect of CP mainly relied on activated FAO by increasing the nuclear localization of PPARα and the expression of CPT1α.

## Materials and methods

### Materials and chemicals

A total of 32 chemical components of the aqueous extract from *Chrysanthemum morifolium* Ramat were detected by the UPLC-MS method, consist of 18 glycosides, 4 flavonoids and their derivatives, 8 phenylpropanoids and 2 other compounds. More details on the ingredients of the aqueous extract of *C. morifolium* flowers was reported in previous publication [17]. The probiotics used in this study was composed of 4 stains including *Bifidobacterium lactis* BPL-1 (10^10^ CFU/g), *Lactobacillus rhamnosus* CNCMl-4036 (10^9^ CFU/g), *Lactobacillus rhamnosus* BPL1-15 (CECT 8361) (10^9^ CFU/g) and *Lactobacillus rhamnosus* GG (10^9^ CFU/g).

### Animal models and experimental design

Male C57BL/6J mice (5-week-old at the beginning of the experiment) were provided by Shanghai Laboratory Animal Center (Shanghai, China). The mice were housed in a specific pathogen-free animal facility with a consistent temperature of 22 ± 1 ℃ and a 12 h light–dark cycle. Before the experiments started, the mice underwent a 7-day period of acclimatization to ensure their health condition. For the CP intervention experiment, the mice were fed with high-fat diet (HFD, with 60% fat, D12492, Research Diet) or chow diet (C2018, SYSEBIO) for 8 weeks. Then the mice fed with HFD were randomly divided into two groups: HFD (450 mg/kg maltodextrin as control, oral gavage daily, *n* = 6) and HFD_CP (100 mg probiotics powder and 900 mg/kg *C. morifolium* extract, oral gavage daily, *n* = 6) for 12 weeks. Maltodextrin accounted for 50% of *C. morifolium* extract. At the same time, other mice were fed with the chow diet group (Control, *n* = 6) for 12 weeks. All animals were euthanized by intraperitoneal injection of pentobarbital sodium at the end of the experiment. All experiments procedures were conducted under the guidelines for Animal Experiment of Shanghai University of Traditional Chinese Medicine and approved by the institutional Animal Ethics Committee (Approval numbers: PZSHUTCM220627032, PZSHUTCM2303060003).

### Microbiota transplantation

For the fecal microbiome transplantation (FMT) experiment, mice were fed with HFD for 5 weeks, followed by a 5-day antibiotic intervention (R_HFD, R_HFD_CP, oral gavage, *n* = 5). The antibiotic intervention regimen was based on previous methods [27]. Concurrent with the antibiotic intervention, donor animals were administered the CP (D_HFD, D_HFD_CP, oral gavage, *n* = 5), followed by a 12-week FMT. Fecal samples were prepared with a three-step centrifugation process. Fresh feces (0.5–0.6 g) were collected daily from donor mice and suspended in 2 ml of clean normal saline. The mixture underwent a series of centrifugations, with the final bacterial precipitate resuspended thoroughly in 2 ml of normal saline.

### Glucose tolerance test (GTT) and insulin tolerance test (ITT)

For the glucose tolerance test (GTT) and insulin tolerance test (ITT), mice were fasted for eight hours and received an intraperitoneal injection (IP) of 1 g/kg glucose for the GTT and 0.75 IU/l insulin for the ITT. Blood glucose levels were measured at 0 min, 15 min, 30 min, 60 min, 90 min, and 120 min after the IP using a glucometer (ACCU-CHEK Performa, Germany).

### Biochemical analysis

Biochemical analysis was conducted on mice euthanized under anesthesia after overnight fasting. Blood samples were collected, separated by centrifugation (1500×*g* for 15 min), and stored at – 80 ℃ for further analysis. Serum alanine aminotransferase (ALT), triglyceride (TG), total cholesterol (TC), aspartate aminotransferase (AST) and low-density lipoprotein (LDL-c) were determined according to the manufacturer's protocols of the appropriate diagnostic kit (Nanjing Jiancheng Bioengineering, C009-2-1, C010-2-1, A112-1-1, A113-1-1, A111-1-1, A110-1-1, China).

### Histological analysis

Liver tissues were fixed in 4% paraformaldehyde for 24 h and embedded in paraffin for hematoxylin and eosin (H&E) staining. Hematoxylin was used for nuclear counterstaining (blue), and eosin was used to stain the cytoplasm red. The degree of hepatic steatosis was evaluated in a blinded manner according to a previous publication. Images of hepatic pathology were observed under a microscope (Eclipse E100, Nikon, Japan). The criteria for hepatic steatosis score were graded as 0 (steatosis involved < 5%), 1 (6% < steatosis involved < 33%), 2 (34% < steatosis involved < 66%), 3 (steatosis involved > 66%).

### 16S rRNA sequencing

Cecal contents of mice were used for analysis of gut microbiota analysis. Fecal DNA was extracted using the QIAamp DNA Stool Mini Kit (Qiagen, Dusseldorf, Germany). The V3–V4 hypervariable region of the bacterial 16S rRNA gene was amplified using the primer pairs 338F (5′-ACTCCTACGGGAGGCAGCAG-3′) and 806R (5'-GGACTACHVGGGTWTCTAAT-3') utilizing an ABI GeneAmp^®^ 9700 PCR thermocycler (ABI, CA, USA). Sequencing was performed based on the published 16S sequencing method [28].

### Western blot analysis

The method for Western blot analysis was derived from previous experimental methods Specifically, Liver tissues were prepared by RIPA buffer supplemented with 1 mM PMSF and phosphatase inhibitors. The nuclear protein of liver tissues was acquired by a Nuclear and Cytoplasmic Protein Extraction kit (Beyotime Institute of Biotechnology, Shanghai, China). All proteins were quantified using the BCA method (20201ES90, Yeasen Biotechnology, Shanghai, China). The primary antibodies are as follows: mouse anti-GAPDH (1:5000, 7076S; Cell Signaling Technology, USA), rabbit anti-CPT1A (1:1000, 15184-1-AP, proteintech, USA), rabbit anti-Fatty Acid Synthase (1:1000, 3180S, Cell Signaling Technology, USA), rabbit anti-PPAR alpha (1:1000, ab215270, ABCAM, USA), rabbit anti-PPAR alpha (1:1000, 15540-1, proteintech, USA), rabbit anti-PGC1 alpha (1:1000, CY6630, Abways, China) and rabbit or mouse secondary antibodies (1:5000, 7076, CST, USA) (1:5000, AS014, ABclonal, China). Appropriate horseradish peroxidase-linked secondary antibodies were used for detection by enhanced chemiluminescence (Share-bio, Shanghai, China).

### Quantitative PCR analysis

RNA from liver tissues was extracted using a Tissue RNA Purification Kit (EZB-RN011A, EZBiscience, Shanghai, China). The cDNA was synthesized at 42 °C for 15 min followed by 95 °C for 30 s using 4 × Reverse Transcription Master Mix (KR118-02, TIANGEN, Beijing, China). Quantitative PCR (qPCR) was then carried out using the SYBR Green Master Mix (Q321-03/03, Vazyme, Nanjing, China). The BioRad CX384 C1000 Thermal Cycler was used to run the qPCR reaction, which involved heating to 95 °C for 3 min, followed by 40 cycles at 95 °C for 15 s then 60 °C for 30 s, and culminating with a melt curve reaction. Primer sets for mouse *18S*, *Cidea*, *Cidec* were used: *18S* (F:5′-CCATCCAATCGGTAGTAGCG-3′, R: 5′-GTAACCCGTTGAACCCCATT-3′; *Cidea* (F:5′-AGCACCTATGCTCCCCGTAA-3′, R: TGGGGCTGGAGTAGCGATTA) *Cidec* (F: 5′-TCGGAAGGTTCGCAAAGGCATC-3′, R: 5′-CTCCACGATTGTGCCATCTTCC-3′).

### Transcriptomics

Total RNA was extracted from liver tissue using Trizol^®^ Reagent according to the manufacturer’s instructions, and genomic DNA was removed using DNase I (Takara, Beijing, China). Subsequently, cDNA libraries were established, and bands of 200–300 bp were screened. Transcriptome sequencing was then performed using the Illumina Hi Seq X/Nova 6000 System, following a published method [29].

### Statistical analysis

All results are presented as mean ± standard error of the mean (SEM). Quantitative image analysis was conducted using ImageJ. Statistical tests such as the two-tailed Student’s *t*-test, Kruskal–Wallis test, Mann–Whitney U, or one-way ANOVA followed by Tukey’s multiple comparisons test were used. Graphpad 8.3.0 was used for graph construction and statistical analysis (GraphPad Software). The correlation between gut microbiota and mouse phenotype was analyzed using Spearman analysis. Significance levels are denoted as follows: *p < 0.05; **p < 0.01; ***p < 0.001.

## Results

### CP alleviates HFD-induced metabolic disorders

**Fig. 1:**
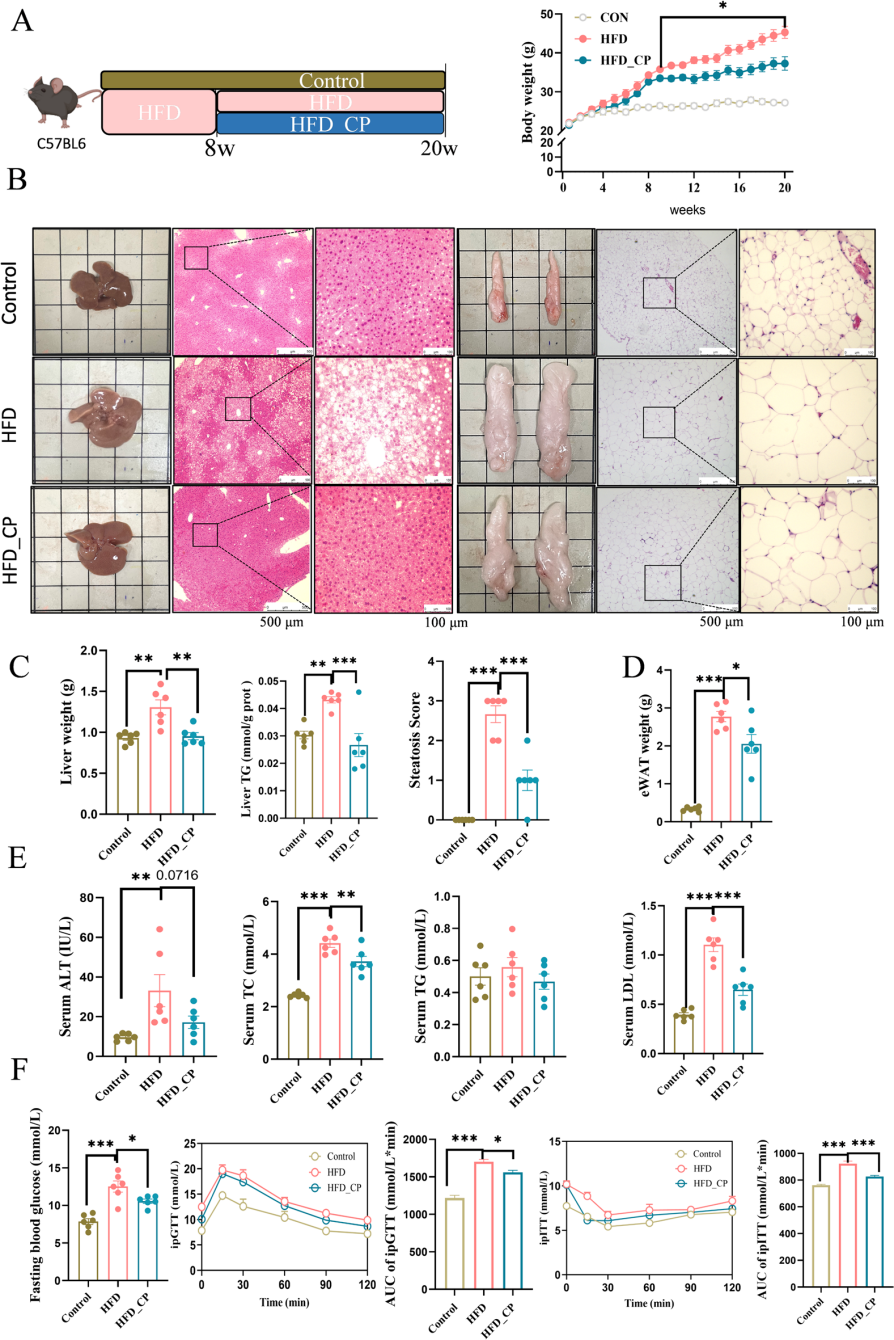
*Chrysanthemum morifolium* Ramat extract and probiotics combination (CP) ameliorates high-fat diet-induced weight gain and liver steatosis. **A** Schematic representation of CP intervention in HFD-fed C57BL/6 mice and body weight change (*n* = 6 per group). **B** Representative images of liver and epididymal adipose tissue as well as H&E staining. **C** Liver weight, TG, and steatosis score. **D** Epididymal adipose tissue (eWAT) weight. **E** Serum ALT, TC, TG level and LDL levels. **F** Fasting blood glucose, intraperitoneal glucose tolerance test (ipGTT), and intraperitoneal insulin tolerance test (ipITT) results with area under the curve (AUC) calculation and. All data are shown as the Mean ± SEM. *p < 0.05, **p < 0.01, ***p < 0.001

Compared with normal chow diet, 20 weeks of a high-fat diet (HFD) feeding significantly increased body weight, liver weight, epididymal fat weight, and hepatic steatosis of mice. A 12-week intervention with CP markedly reduced body weight gain and fat accumulation in the liver and adipose tissue, as evidenced by liver triglyceride (TG) level and hematoxylin and eosin (H&E) staining (Fig. 1A–D). In addition, the elevated levels of serum ALT, TC, and LDL induced by HFD were reduced after CP administration (Fig. 1E). Moreover, CP significantly regulated host glucose homeostasis. CP reduced the fasting blood glucose level of HFD-fed mice, enhanced glucose tolerance, and alleviated the insulin resistance in HFD-fed mice (Fig. 1F). Together, these results suggest that CP effectively ameliorated HFD-induced metabolic disorders.

### CP regulates gut microbiota composition in HFD-fed mice

**Fig. 2:**
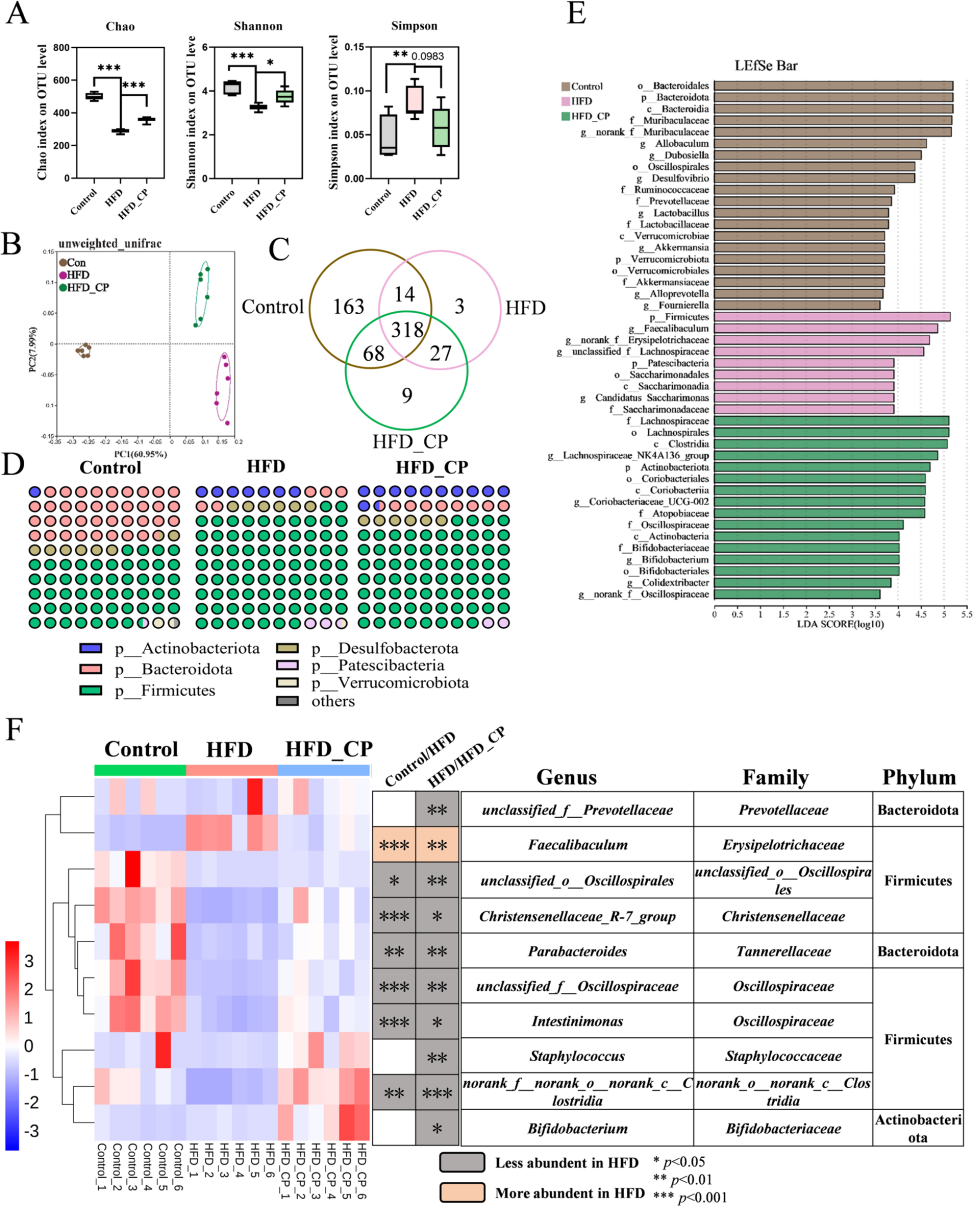
CP regulates gut microbiota composition in HFD-fed mice. **A** Alpha diversity analysis (Chao index, Shannon index and Simpson index) on OUT level. **B** Beta diversity analysis using the unweighted unifrac method. **C** Venn plot of the number of OTUs in the control, HFD, and HFD_CP. **D** Relative abundance of bacteria at phylum level. **E** Linear discriminant analysis effect size (LEfSe) analysis of the characteristic genera of the gut microbiota. Distribution histogram based on LDA; a higher LDA score represents greater importance of the bacteria. **F** Heatmap showing the top 10 genera significantly altered by CP in HFD-fed mice. All data are shown as the Mean ± SEM. *p < 0.05, **p < 0.01, ***p < 0.001

It is well known that the disorders of glucose and lipid metabolism caused by HFD are closely related to the gut microbiota. We next investigated the changes of gut microbiota composition and function after CP intervention. At the operational taxonomic unit (OTU) level, the reduced Chao and Shannon indexes and increased Simpson index caused by HFD feeding were restored by CP treatment, suggesting that α diversity was increased (Fig. 2A). The results of principal coordinate analysis (PCoA) showed that CP was separated from the HFD and control group, but was close to the control group at the PC1 axis. The Venn plot indicated that the decline of OTU caused by HFD was improved by CP (Fig. 2B, C). At the phylum level, CP increased the relative abundance of Actinobacteriota and Bacteroidota, but reduced the relative abundance of Firmicutes in HFD-fed mice (Fig. 2D). Linear discriminant analysis-effect size (LEfSe) analysis was used to identify characteristic bacteria in each group. Consistently, the HFD group had a relatively higher abundance of the Firmicutes phylum and *Faecalibaculum* genus. In contrast, CP group mainly increased the abundance of genera belonging to *Lachnospiraceae*, *Oscillospiraceae*, and *Bifidobacteriaceae* families (Fig. 2E). Therefore, we next performed a TOP10 heatmap analysis with significant changes in the CP group at the genus level. The relative abundance of genus *Faecalibacterium* under Firmicutes was significantly higher in HFD group but was reduced with CP intervention. In addition, CP significantly reversed HFD-induced reduction of *unclassified_o_Oscillospirales*, *Christensenellaceae_R-7_group*, *Parabacteroides*, *unclassified_f_Oscillospiraceae*, *Intestinimonas*, and *norank_f_norank_o_norank_c_Clostridia*, suggesting the markedly alternation of gut microbiota after CP intervention (Fig. 2F). Moreover, the relative abundance of *Lactobacillus rhamnosus* and *Bifidobacterium* were higher in CP group, which indicated a successful localization of these probiotics after CP treatment (Fig. S1).

**Fig. 3:**
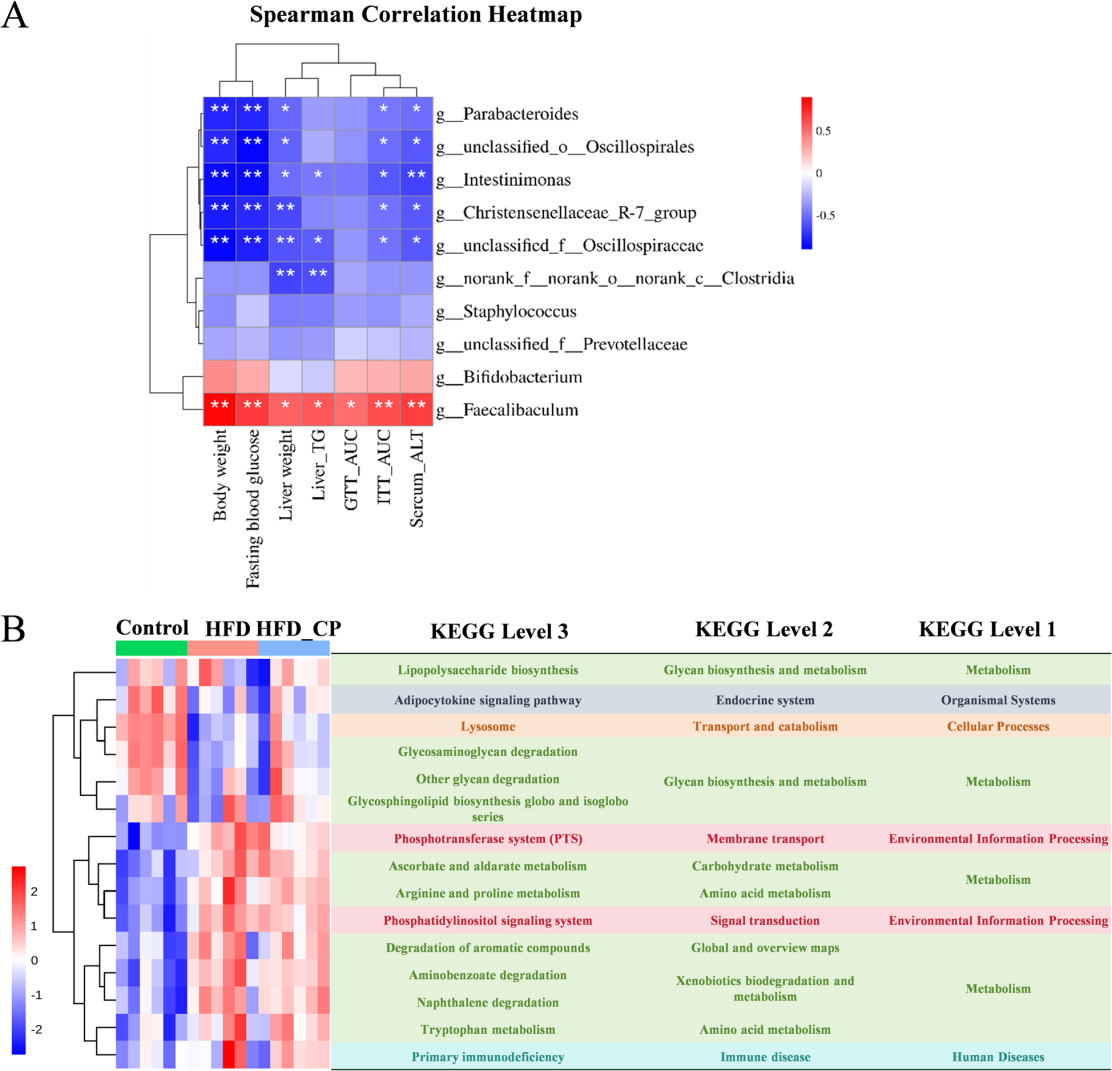
The correlation of bacteria with mouse phenotype and function predication. **A** Correlation analysis of the gut microbiota and mouse phenotypes in three groups. **B** The prediction of gut microbial function based on 16S rRNA sequencing by PICRUSt2. *p < 0.05, **p < 0.01

To demonstrate the correlation between alterations in gut microbiota and disorders in glucose and lipid metabolism in mice, we conducted a Spearman correlation analysis using the aforementioned 10 genera and indicators related to lipid and glucose metabolism (Fig. 3A). Out of the 10 genera, five, including *Parabacteroides*, *unclassified_o_Oscillospirales*, *Intestinimonas*, *Christensenellaceae_R-7_group*, *unclassified_f_Oscillospiraceae*, exhibited a negative correlation with body weight, liver weight, fasting blood glucose level, ITT_AUC, and serum ALT levels. However, *Faecalibaculum* was positively related to these indicators as well as liver TG and GTT_AUC. The structure changes of gut microbiota were always accompanied by the alternation of function. Thus, the functional changes in gut microbiota following CP intervention were predicted using PICRUSt2 analysis based on the results of 16S rRNA sequencing. Among the top 15 altered pathways, 4 pathways were related to glycan biosynthesis and metabolism, 2 pathways were related to amino acid metabolism (Fig. 3B). These findings suggest that CP intervention regulated gut microbiota composition and function in HFD-fed mice, which might play an essential role in improving metabolic disorders.

### CP improves glucose homeostasis partly through regulating gut microbiota

**Fig. 4:**
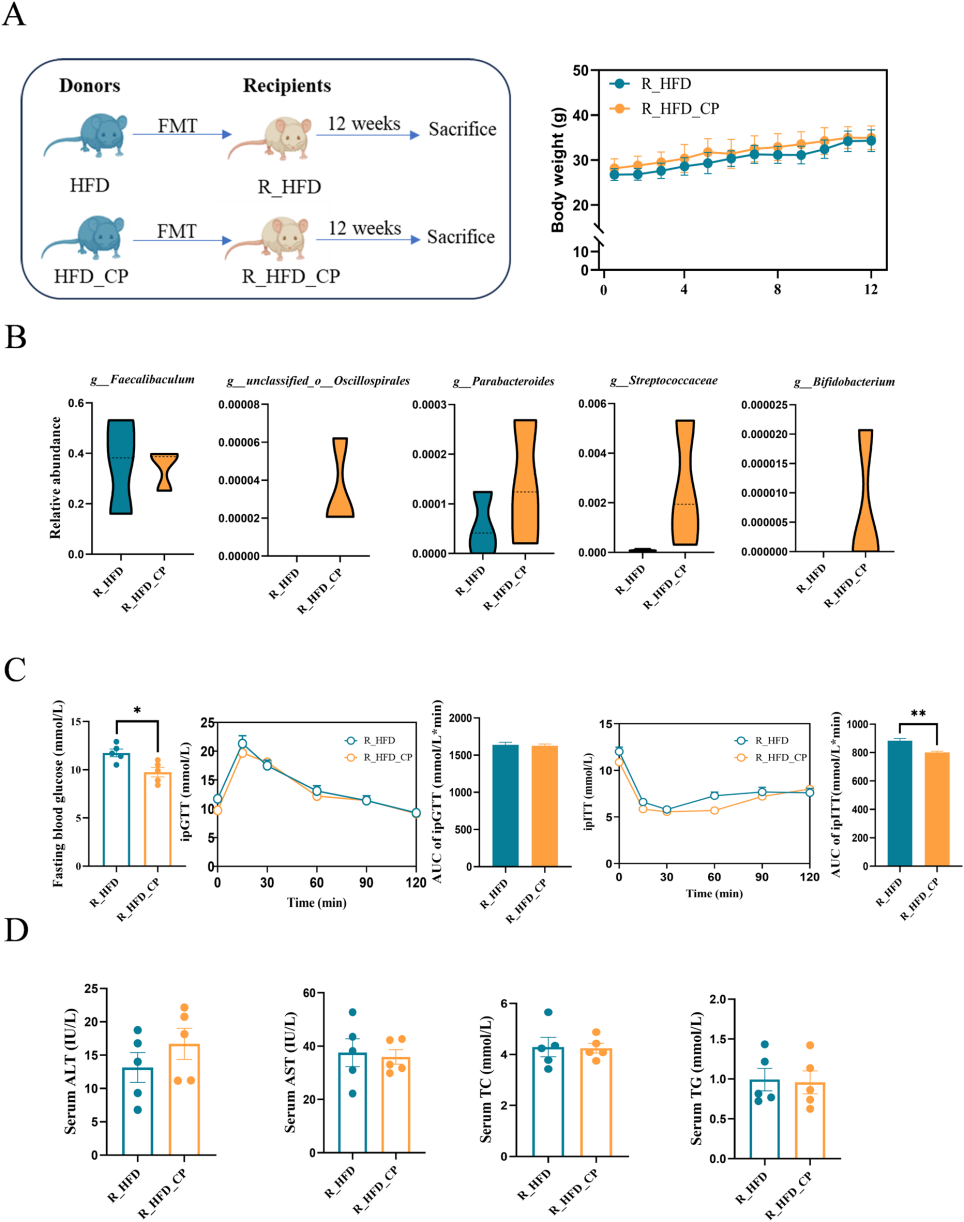
Glucose metabolism is improved by fecal transplantation from CP-treated mice to HFD-fed mice. **A** Schematic diagram of FMT experiment and body weight (*n* = 5 per group). **B** The relative abundance of representative genus in the recipient mice. **C** Fasting blood glucose, intraperitoneal glucose tolerance test (ipGTT), and intraperitoneal insulin tolerance test (ipITT) results with area under the curve (AUC) calculation. **D** Serum ALT, AST, TC, and TG levels. All data are shown as the Mean ± SEM. *p < 0.05, **p < 0.01

To test whether ameliorating metabolic disorders by CP is dependent on gut microbiota, we conducted fecal microbiota transplantation (FMT) experiment (Fig. 4A). We observed no difference in body weight among the recipient mice. But post-FMT, the relative abundance of *g_unclassified_o_Oscillospirales*, *g_Parabacteroides*, *g_ Streptococcaceae*, and *g_Bifidobacterium* were higher in the R_HFD_CP mice than in the R_HFD mice (Fig. 4B), aligning with the trends observed in the donor (Fig. 2F). This suggests successful colonization of fecal bacteria. Further GTT and ITT experiments showed that the fecal microbiota from HFD_CP group did not improve glucose tolerance but significantly reduced fasting blood glucose level and improved insulin resistance (Fig. 4C). The serum biochemical analysis indicated that FMT from HFD_CP mice did not improve lipid metabolism or liver injury-related indices compared with the mice received FMT from HFD mice (Fig. 4D). These data suggest that CP-altered gut microbiota play an important role in regulating host’s glucose metabolism.

### CP regulates hepatic expression profile, particularly lipid metabolism-related pathways

**Fig. 5:**
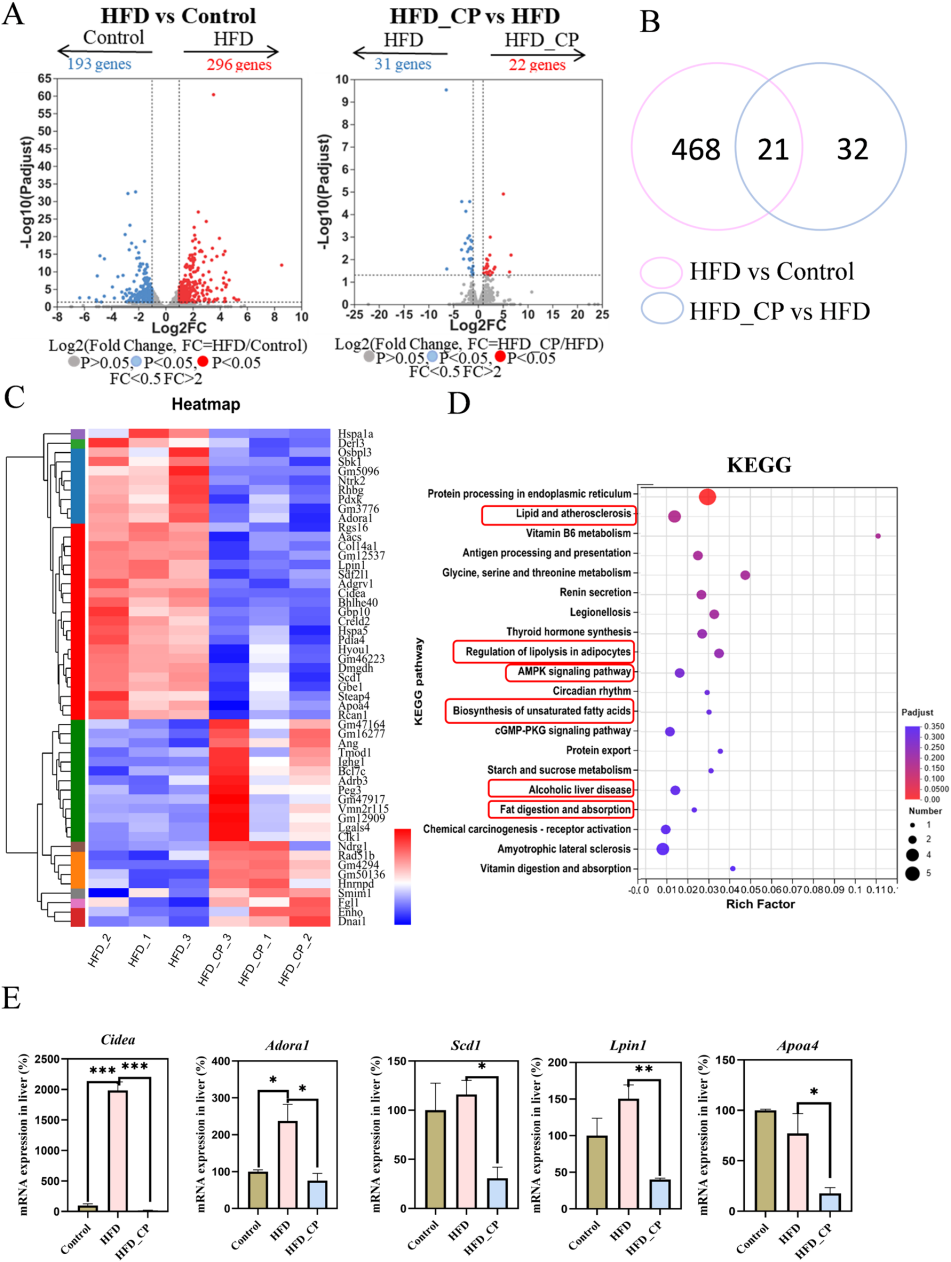
Hepatic transcriptomic analysis of livers from CP treated mice. Mice were treated as in Fig. 1. **A** Volcano plot based on the changed genes of the HFD group compared with the Control group; the HFD_CP group compared with the HFD group. **B** Venn diagram of regulated genes by HFD or by HFD_CP. **C** Heatmap of the significantly changed genes between HFD and HFD_CP groups. **D** KEGG enrichment analysis of the reversed genes by CP. **E** The changes of lipid metabolism-related genes reversed by CP based on hepatic transcriptomic analysis. All data are shown as the Mean ± SEM. *p < 0.05, **p < 0.01, ***p < 0.001

Because gut microbiota only partly explained the improvement of glucose metabolism by CP intervention, to identify the possible anti-NAFLD mechanism of CP, we conducted RNA sequencing (RNA-seq) analysis of liver tissue from three group of mice. The result indicated that 296 genes were upregulated and 193 genes were downregulated in HFD group compared with Control group. In addition, when compared to HFD group, there were 22 genes upregulated and 31 genes downregulated (fold change > 2 or < 0.05, adjusted *p* < 0.05), respectively (Fig. 5A). Among them, 21 genes were commonly altered by HFD and CP (Fig. 5B). The heatmap revealed significant changes of 53 genes between HFD and HFD_CP groups (Fig. 5C). KEGG pathway analysis of altered genes between HFD_CP and HFD groups revealed distinct shifts in metabolic pathways. Specifically, CP intervention regulated numerous lipid metabolism-related pathways, including lipid and atherosclerosis, regulation of lipolysis in adipocytes, AMPK signaling pathway, biosynthesis of unsaturated fatty acids, alcoholic liver disease and fat digestion and absorption (Fig. 5D). We noticed that the expression of *Cidea* and *Scd1* that belonging to AMPK signaling pathway, as well as *Aodra1*, *Lpin1*, and *Apoa4* that belonging to lipid metabolism related pathways were reduced in HFD_CP group than HFD group based on RNA-seq results (Fig. 5E).

**Fig. 6:**
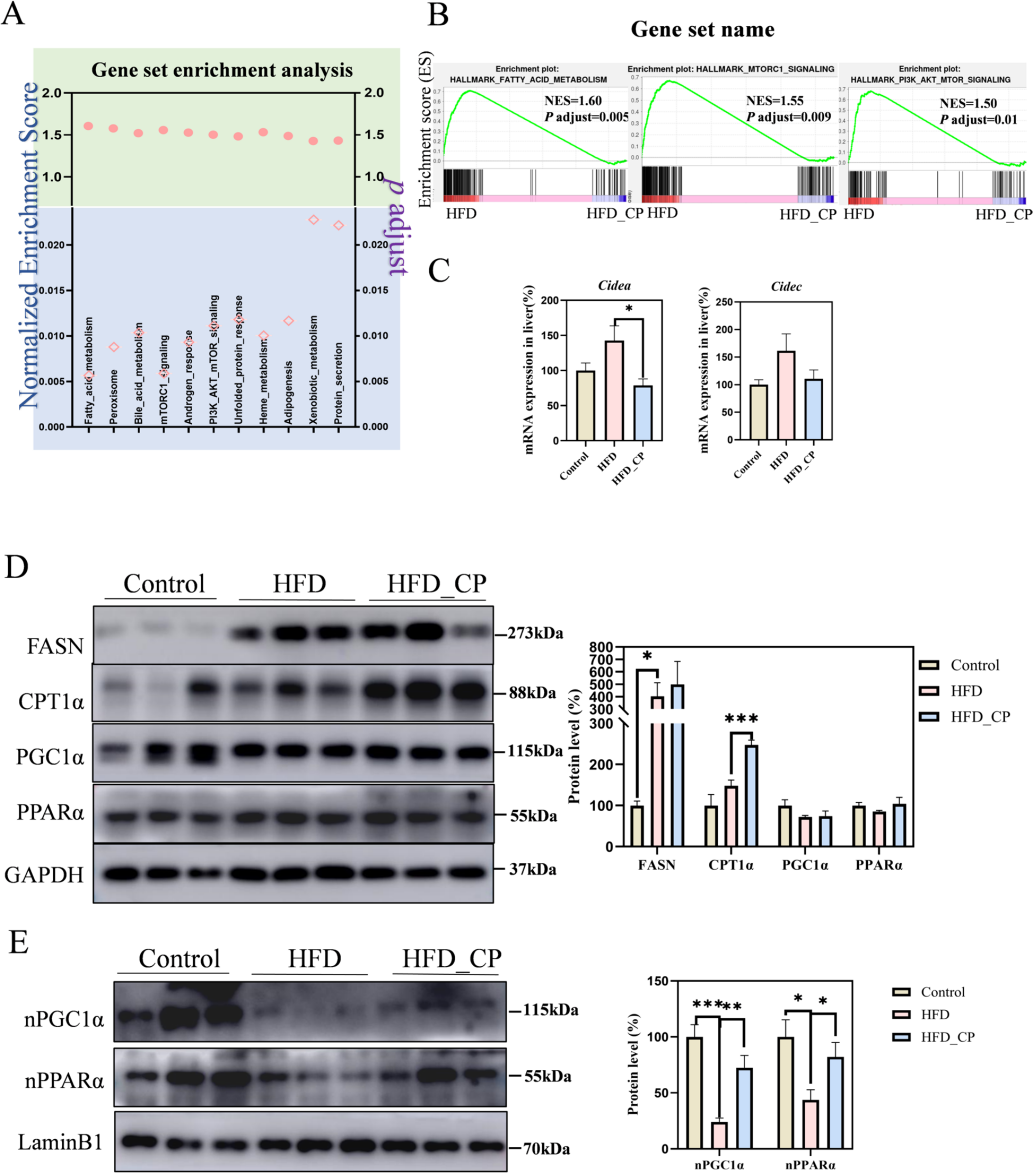
CP improves HFD-induced NAFLD by activating PPARα. **A** The reversed pathways by CP, which was enriched with gene set enrichment analysis (GSEA) (adjusted *p* < 0.05). **B** The fatty acid metabolism, mTORC1 signaling, and PI3K-AKT-mTOR signaling pathways which are from the GSEA analysis of the liver gene profiling. Mice were treated as in Fig. 1. **C** mRNA expression of *Cidea* and *Cidec* in the liver. **D** The expression of total proteins of FASN, PPARα, PGC1α and CPT1α in the liver. **E** The protein level of nuclear PPARα and nuclear PGC1α in the liver. All data are shown as the Mean ± SEM. *p < 0.05, **p < 0.01, ***p < 0.001

In order to elucidate the pathways regulated by CP more comprehensively, we performed Gene Set Enrichment Analysis (GSEA) of all genes. Based on the NES score and *P* adjust value, the fatty acid metabolism (core enrichment genes including *Cidea*, *Cd36*, *Fasn*, *Acox1*, et al*.*), mTORC1 signaling (core enrichment genes including *Hspa5*, *Sdf2l1*, et al*.*), and PI3K-AKT-mTOR signaling pathways (core enrichment genes including *Akt1*, *Pten*, *Pik3r3*, et al*.*), which are closely related to NAFLD formation and development, were found to be related to CP treatment (Fig. 6A, B). Based on the above analysis, we speculate that CP improves NAFLD possibly by regulating lipid metabolism-related pathways.

### CP improves HFD-induced NAFLD by facilitating PPARα nuclear localization

Based on the above RNA-seq results, we found that the expression of *Cidea* gene was particularly significantly reduced by CP. The CIDE family protein (including CIDEA, CIDEB and CIDEC) is a class of lipid droplet-binding proteins that are mainly enriched at the lipid droplet contact sites (LDCS), promoting the storage of neutral lipids in cells, and plays an important role in lipid homeostasis [30]. We further performed RT-qPCR analysis to investigate their expression. Our results showed that CP intervention downregulated the expression of *Cidea* and *Cidec* in the liver, which was consistent with the trend of RNA-seq result (Fig. 6C). Fatty acid synthase (FASN) and CPT1α are the key enzymes responsible for the de novo synthesis of fatty acids and FAO, respectively. We first analyzed their protein levels in the liver of CP treated mice by Western blot. The results showed that CP intervention had little effect on affecting the expression of FASN, but significantly upregulated the expression of CPT1α. Because PPARα is the core transcription regulator for FAO and *Cidea* and *Cidec* are the target genes of PPARα [30, 31], we studied the total protein levels of PPARα and transcription coactivator PGC1α. However, CP intervention didn’t affect the total levels of these two proteins (Fig. 6D). Nuclear localization is the prerequisite for transcriptional regulators to regulate gene expression. Thus, we further studied the nuclear localization of PPARα and PGC1α. Surprisingly, HFD reduced the protein levels of PPARα and PGC1α in the nucleus, while CP intervention significantly restored their nuclear localization to the level of control group (Fig. 6E). Altogether, these findings suggest that CP promotes the nuclear localization of PPARα leading to increased expression of CPT1α and activated FAO, as well as decreased expression of *Cidea* and *Cidec* and more substrates for fatty acid β-oxidation.

### The anti-NAFLD effect of CP is PPARα-dependent

**Fig. 7:**
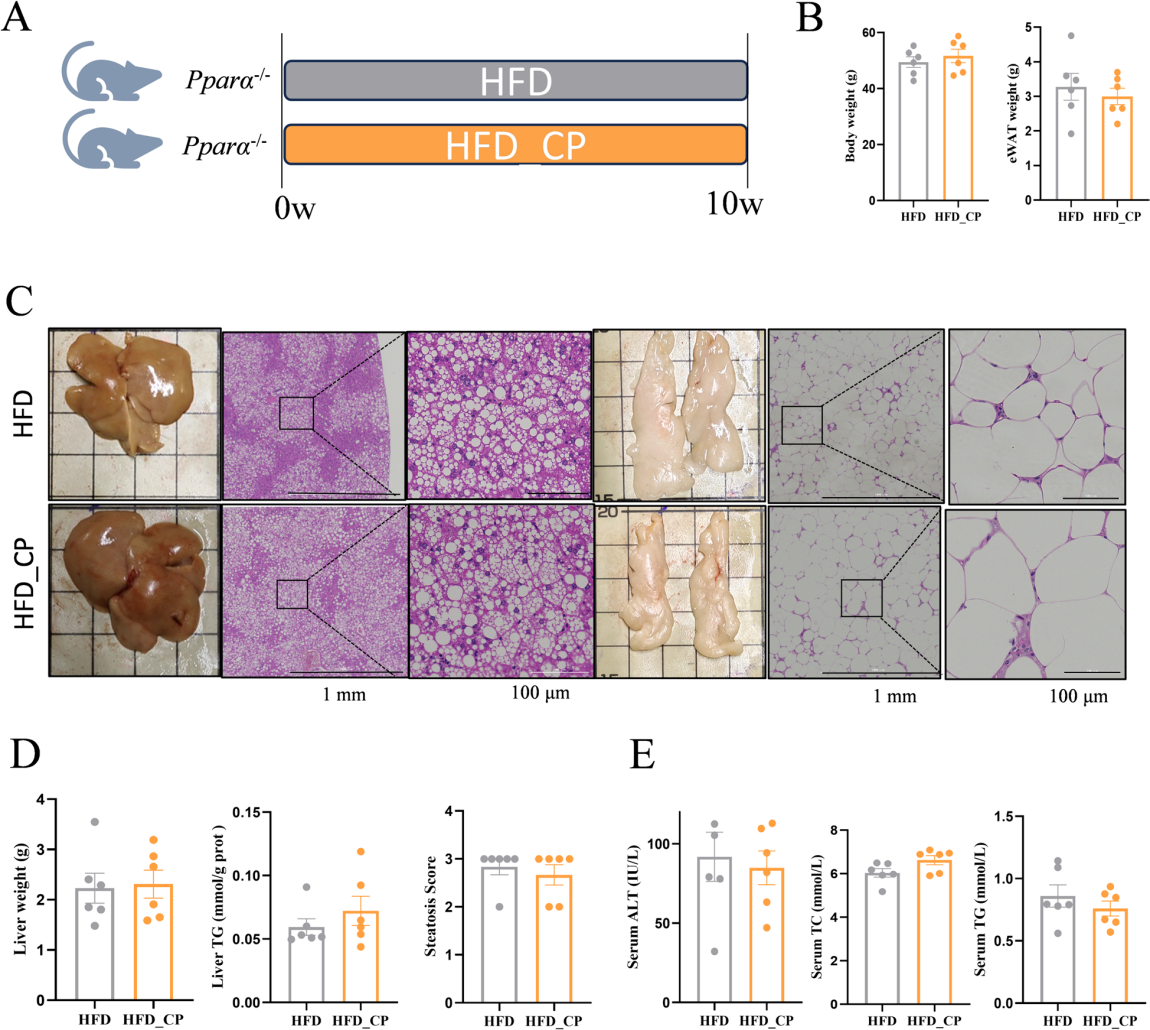
PPARα deficiency abolishes HDCA-mediated anti-NAFLD effect. **A** Schematic representation of CP intervention in HFD-fed *Pparα*^−/−^mice (n = 6 per group). **B** Body weight and epididymal adipose tissue (eWAT) weight. **C** Representative images of liver and epididymal adipose tissue as well as H&E staining. **D** Liver weight, TG, and steatosis score. **E** Serum ALT, TC, TG level

To further investigate whether the anti-NAFLD effect of CP is PPARα-dependent, we constructed whole-body *Pparα* knockout (*Pparα*^−/−^) mice (Fig. S2). CP intervention failed to improve NAFLD-related parameters in *Pparα*^−/−^ mice such as body weight, epididymal adipose weight, liver TG, hepatic steatosis score (Fig. 7A–D). The serum biochemical analysis indicated that CP mice did not improve ALT, serum TC or TG compared with HFD mice (Fig. 7E). These results demonstrate that PPARα is essential for the anti-NAFLD effect of CP.

## Discussion

**Fig. 8:**
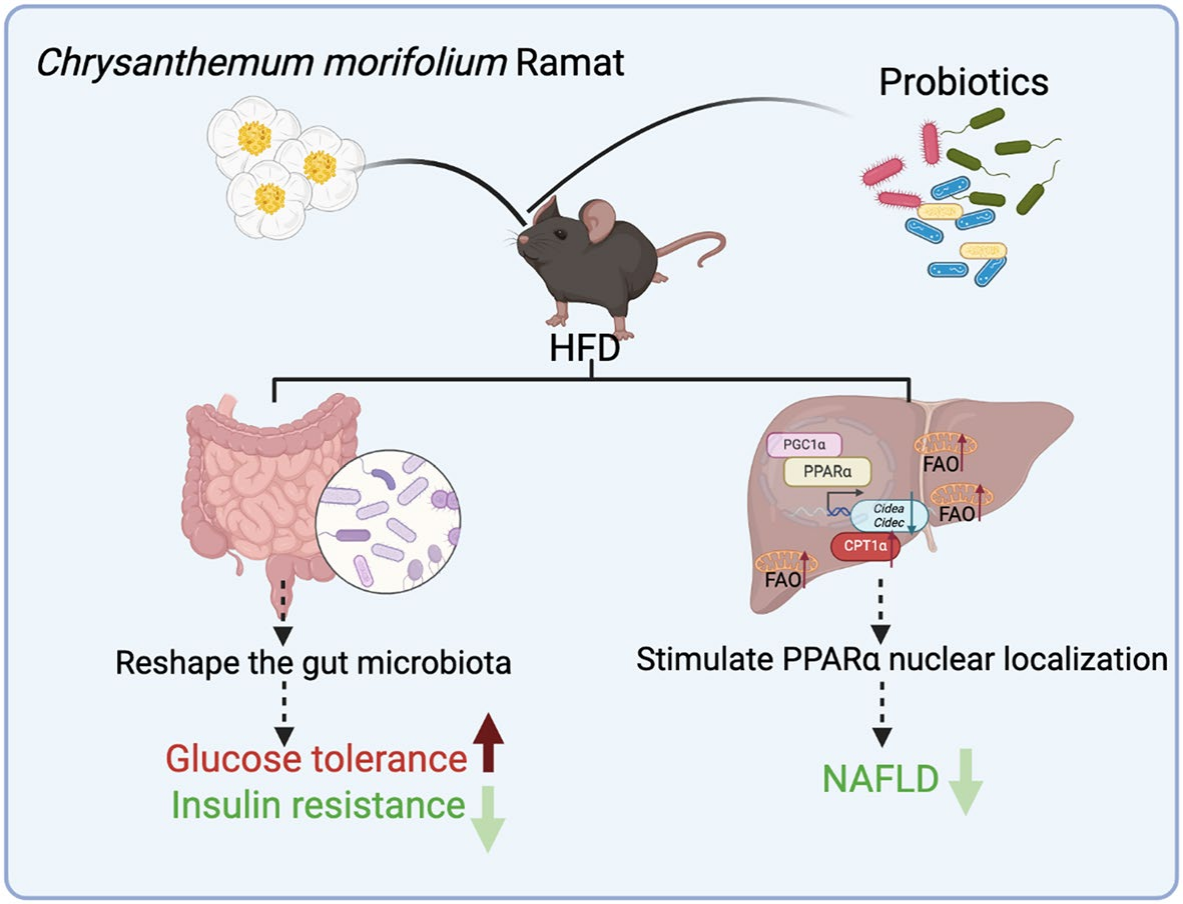
Schematic diagram of the potential mechanisms of CP on ameliorating metabolic disorders

In this study, we found that CP intervention alleviated HFD-induced glucose metabolism disorder and hepatic steatosis. To explore the underlying mechanism, we studied the gut microbiota composition and found that probiotics could colonize successfully under *C. morifolium* intervention. FMT result indicated that CP played an important role in improving glucose metabolism through regulating the composition and function of gut microbiota. Additionally, the anti-NAFLD effect of CP was PPARα-dependent. CP stimulated the nuclear localization of PPARα protein, increased the protein expression of downstream CPT1α, and downregulated the gene expression of *Cidea* and *Cidec,* thus leading to the activation of FAO in the liver (Fig. 8).

Previous studies have shown that the gut-liver axis played a vital role in progression of many diseases, such as NAFLD and T2DM [32]. HFD-fed germ-free mice do not develop obesity-related phenotypes, indicating the importance of gut microbiota on regulating host metabolism [33]. *Bifidobacteria* and *Lactobacillus rhamnosus* have been shown to have beneficial functions for human health like anti-cancer, immune regulation, and so on [34, 35]. Recently, Wang et al*.* discovered that certain *Bifidobacteria* and *Lactobacillus* strains could alleviate diet-induced NAFLD in mice through modulation of various gut microbiota-dependent pathways [36]. Similarly, as a natural plant and traditional Chinese medicine, *C. morifolium* is widely used in our daily life and is beneficial to human health in many aspects. It has been proved to be clinically effective for a wide variety of diseases such as fever, headache, sore throat, hypertension, etc. [37]. Previous in vitro study found that ethanol extract from *C. morifolium* flowers inhibited the growth of certain pathogenic bacteria but moderately promoted the growth of commensal probiotics such as *Lactobacillus* and *Bifidobacterium* [16]. A further study demonstrated that oral administration of polysaccharides from *C. morifolium* could be an effective treatment for ulcerative colitis. This was achieved by increasing the diversity of intestinal flora, rising Firmicutes/Bacteroidetes ratio, and promoting the growth of beneficial intestinal flora in rats [38]. Chlorogenic acid is one of the important compounds in *C. morifolium* extract. It has been shown that chlorogenic acid improved NAFLD by regulating gut microbiota [39]. In addition, another compound from *C. morifolium* extract, apigenin, could alleviate obesity-associated metabolic syndrome by regulating gut microbiota and restoring gut barrier damage [40]. In current study, we first analyzed the effect of *C. morifolium* aqueous extract on the colonization of *Lactobacillus* and *Bifidobacterium* in mice and found these two kinds of probiotics could successfully colonize in mouse intestine. CP combination treatment significantly affected the structure and function of gut microbiota in mice. For example, at the genus level, CP group showed significant increased abundance of *Parabacteroides* and *Oscillospirales* while a significant decreased abundance of *Faecalibacterium* compared with the HFD group. Previous study found that the relative abundance of *Faecalibacterium* was positively correlative with the development of T2DM, while *Parabacteroides* abundance showed a negative correlation [41, 42]. *Oscillospirales,* a genus capable of producing short-chain fatty acids (SCFAs) such as butyrate, is also listed as a candidate for the next generation of probiotics [43]. In the correlation analysis of this study, *Oscillospirales* was found to be negatively correlated with mouse blood glucose-related indicators and liver TG. Consistent with other publications, our results showed the similar relationship between these three genera and metabolic status after CP administration. Additionally, the function of gut microbiota after CP intervention was altered, especially for the glycan biosynthesis and metabolism-related pathway. These findings suggested that the changes of gut microbiota by CP intervention might be close related to glucose metabolism. Thus, we further performed FMT experiment to investigate the role of CP-induced changes of gut microbiota on host metabolism. The result showed that certain bacteria changed by CP in the donor can be transferred to the recipient mice, and fecal bacteria from CP-treated group reduced fasting blood glucose level and insulin resistance in the recipients, indicating CP improved glucose homeostasis partly through regulating gut microbiota. However, fecal bacteria from CP-treated group did not improve lipid metabolism or liver injury-related indices compared with the mice received FMT from HFD mice, which suggested that CP induced improvement of lipid metabolism and liver function might not all dependent on gut microbiota, and some other mechanisms such as interaction between *C. morifolium* and probiotics might be involved.

The liver is an essential organ for lipid metabolism. Hepatic lipid homeostasis are regulated through four main pathways: uptake of circulating lipids, de novo lipogenesis, FAO, and lipid output in very low-density lipoprotein, which are closely regulated by complex interactions between hormones, nuclear receptors, and transcription factors [44]. Studies have found that agonists of PPARα enhance insulin sensitivity, glucose homeostasis, lipid metabolism, and exert anti-inflammatory effects [45]. In mammalian cells, PPARα is essential for the regulation of fatty acid oxidation, which occurs mainly in mitochondria [44]. An increased level of PPARα and PGC1α in the nucleus initiated the increased expression of downstream FAO-related genes, thereby alleviated lipid accumulation. CPT1α, located on the outer membranes of mitochondria, is the rate-limiting enzyme for FAO [46]. Previous study showed that ethanol extract of *C. morifolium* leaves attenuated hepatic steatosis in diet-induced mice model by downregulating the mRNA expression of lipogenic genes (*Srebf1c*, *Srebf2*, *Fasn*, *Scd1*, and *Acaca*) and upregulating *Cpt1α* [14], and chlorogenic acid attenuated fatty liver by up-regulating the gene expression of PPARα in diet-induced hypercholesterolemic rats [47]. However, our results found that the aqueous extract of *C. morifolium* flowers and probiotics combination did not affect de novo lipogenesis or the total protein levels of PPARα or PGC1α. Surprisingly, the nuclear amount of PPARα and PGC1α was significantly increased by CP, leading to the elevated expression of CPT1α in the liver tissue and activated FAO in mitochondria.

Accumulation of TG in lipid droplets (LDs) is the main cause of hepatic steatosis. LDs are organelles for fat storage, providing high-energy substrates for FAO in mitochondria and peroxisomes [48]. The CIDE family play important roles in lipid droplet formation. CIDEA and CIDEC are LD-associated proteins that promote lipid storage in adipocytes [49]. Overexpression of *Cidea* in mouse liver resulted in increased hepatic lipid accumulation and the formation of large LDs [25]. Mice with *Cidea* deficiency had decreased lipid accumulation and alleviated hepatic steatosis when they received an HFD feeding [25]. Adenoviral-mediated silencing of hepatic *Fsp27/Cidec* abolishes fasting-induced liver steatosis in the absence of changes in plasma lipids [30]. PPARα is not only the core transcription regulator for FAO but also controls the expression of *Cidea* and *Cidec* in the liver [50]. The whole-body knockout of *Pparα* promoted the development of NAFLD [51]. CP intervention reversed HFD-induced expression of *Cidea*/*Cidec* based on RNA-seq and/or RT-qPCR results, which might lead to less formation of LDs in liver. Additionally, our results showed that the anti-NAFLD effect of CP was abolished in whole-body *Pparα* knockout mice. Therefore, our results demonstrate that CP mitigated the development of NAFLD through the PPARα mediated FAO pathway and LDs formation.

## Conclusion

In summary, this study shows that CP alleviates the glucose and lipid metabolism in HFD-fed mice through regulating gut microbiota composition and hepatic subcellular localization of PPARα, respectively. Our current study highlights the potential of CP supplement (prebiotics and probiotics combination) as a functional food for the prevention and treatment of metabolic disorders. Further investigations are warranted to elucidate the material basis of the *C. morifolium* extract that contributes to its beneficial effects.

### Supplementary Information

Supplementary Material 1.

## Data Availability

All data generated or analyzed during this study are included in this published article.

## Abbreviations

NAFLD: Nonalcoholic fatty liver disease
T2DM: Type 2 diabetes mellitus
NASH: Nonalcoholic steatohepatitis
FMT: Fecal microbiota transplantation
TCM: Traditional Chinese Medicine
CP: *C. morifolium* Extract and probiotics combination
PPARα: Peroxisome proliferators activate receptor α
PGC1α: Peroxisome proliferator-activated receptor γ coactivator 1 − α
CPT1α: Carnitine palmitoyl transferase 1α
CIDE: DNA fragmentation factor 45-like effector
FAO: Fatty acid oxidation
HFD: High-fat diet
GTT: Glucose tolerance test
ITT: Insulin tolerance test
ALT: Alanine aminotransferase
TG: Triglyceride
TC: Total cholesterol
AST: Aspartate aminotransferase
LDL-c: Low-density lipoprotein
H&E: Hematoxylin and eosin
OUT: Operational taxonomic unit
PCoA: Principal coordinate analysis
LEfSe: Linear discriminant analysis-effect size
RNA-seq: RNA sequencing
GSEA: Gene Set Enrichment Analysis
LDCS: Lipid droplet contact sites
FASN: Fatty acid synthase
DNL: De novo lipogenesis
VLDL: Very low-density lipoprotein
FA: Fatty Acyls
LDs: Lipid droplets
