# Supplementary figures and images for: Chrysanthemum morifolium Ramat extract and probiotics combination ameliorates metabolic disorders through regulating gut microbiota and PPARα subcellular localization
Source: Chin Med. 2024 Jun 3;19:76. doi: 10.1186/s13020-024-00950-w (PMC11149226; doi:10.1186/s13020-024-00950-w)

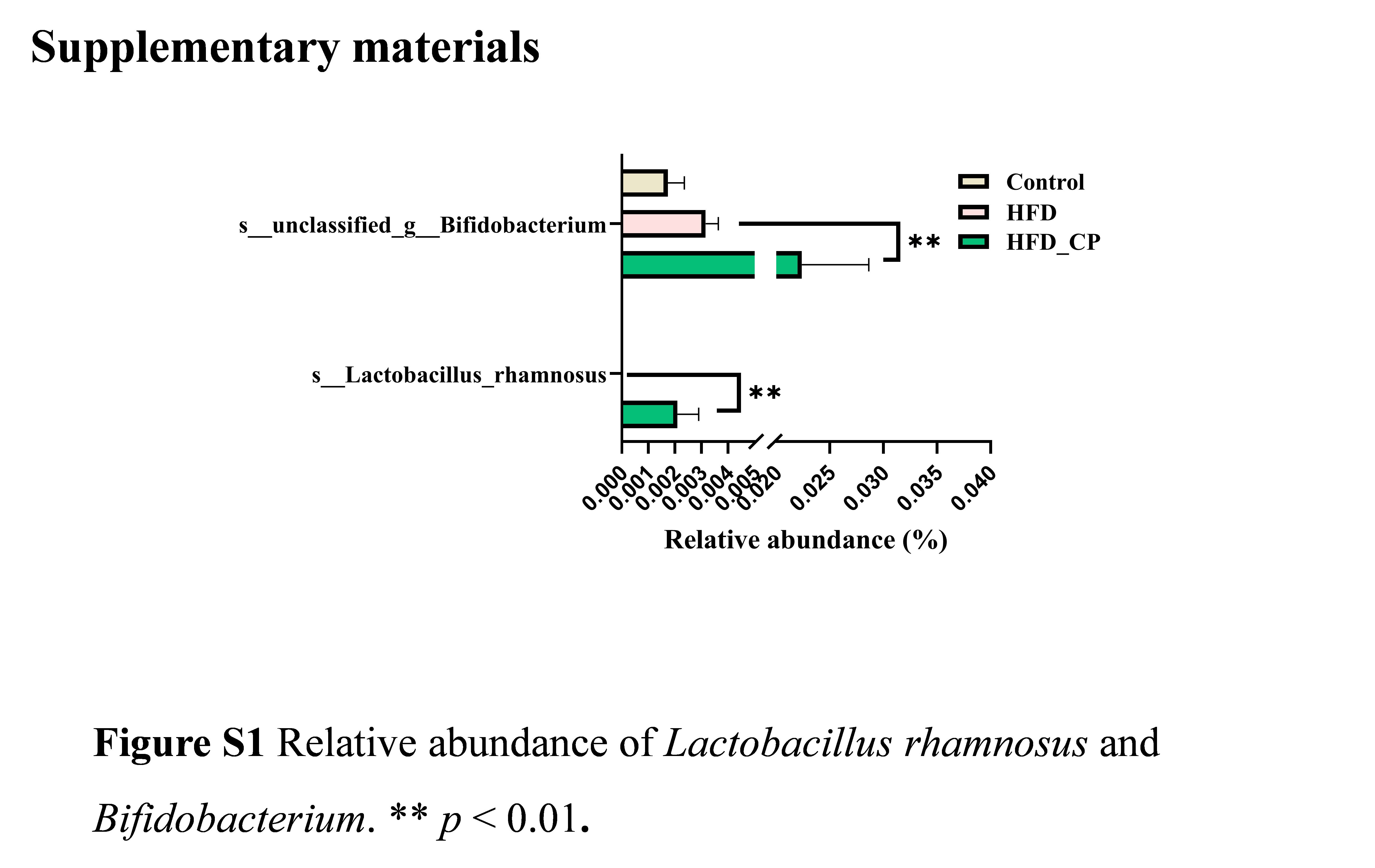

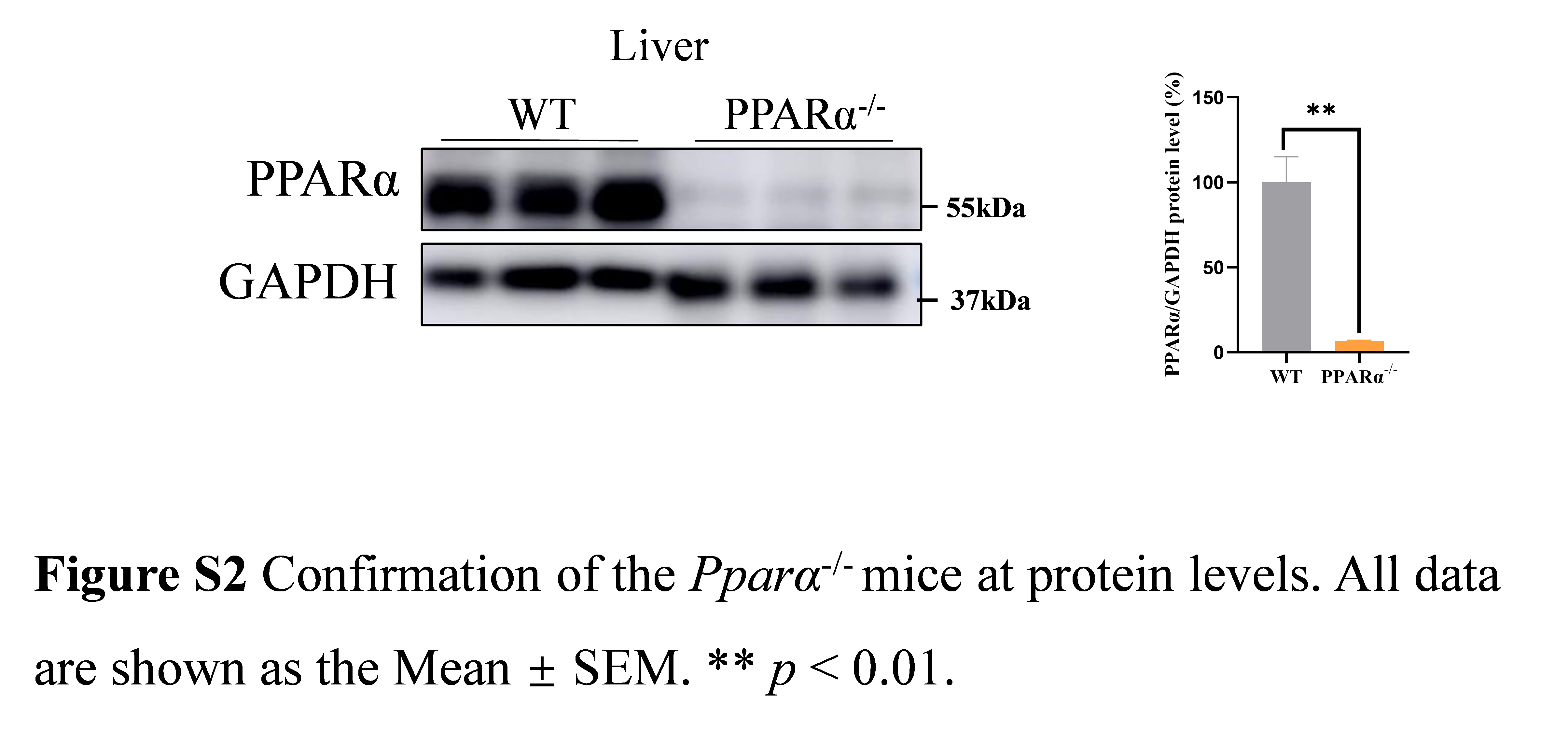

Supplement: Supplementary file 1 — Supplementary Material 1. [file 13020_2024_950_MOESM1_ESM.docx]
